# Birth cohort differences in cognitive performance in 75- and 80-year-olds: a comparison of two cohorts over 28 years

**DOI:** 10.1007/s40520-020-01702-0

**Published:** 2020-09-12

**Authors:** Matti Munukka, Kaisa Koivunen, Mikaela von Bonsdorff, Sarianna Sipilä, Erja Portegijs, Isto Ruoppila, Taina Rantanen

**Affiliations:** Faculty of Sport and Health Sciences and Gerontology Research Center, University of Jyväskylä, P.O. Box 35, 40014 Jyväskylä, Finland

**Keywords:** Cognitive performance, Digit span test, Digit symbol test, Verbal fluency test, Reaction time

## Abstract

**Objective:**

To evaluate cohort differences in cognitive performance in older men and women born and assessed 28 years apart.

**Methods:**

Data in this study were drawn from two age-homogeneous cohorts measured in the same laboratory using the same standardized cognitive performance tests. Participants in the first cohort were born in 1910 and 1914 and assessed in 1989–1990 (Evergreen project, *n* = 500). Participants in the second cohort were born in 1938 or 1939 and 1942 or 1943 and assessed in 2017–2018 (Evergreen II, *n* = 726). Participants in both cohorts were assessed at age 75 and 80 years and were recruited from the population register. Cognitive performance was measured using the Digit Span test from the Wechsler Memory Scale (WMS), Digit Symbol test from the Wechsler Adult Intelligence Scale (WAIS) and phonemic Verbal Fluency test from the Schaie-Thurstone Adult Mental Abilities Test. Reaction time assessing motor and mental responses was measured with a simple finger movement task, followed by a complex finger movement task. T-tests were used to study cohort differences and linear regression models to study possible factors underlying differences.

**Results:**

We found statistically significant cohort differences in all the cognitive performance tests, except for the digit span test and simple movement task in men, the later-born cohort performing better in all the measured outcomes.

**Conclusions:**

The results of this study provide strong evidence that cognitive performance is better in more recent cohorts of older people compared to their counterparts measured 28 years earlier.

## Introduction

Aging is associated with a general decline in cognitive performance, such as memory, attention, processing speed and problem solving, and affects the way people learn and perform [1]. The “Flynn effect” [2], i.e., later-born cohorts outperforming earlier born cohorts in cognitive abilities, has been observed for older people when measured at the same chronological ages [3]. Based on evidence from standardization samples of the Wechsler Intelligence Scale for Children (WISC) tests, young Americans gained about 22 IQ points (where 15 IQ points represent a standard deviation) over the 70 years between 1932 and 2002 [4]. Analyses of the new Wechsler Adult Intelligence Scale–Fourth Edition (WAIS–IV) suggest a continued and steady annual mean increase in IQ of approximately 0.3 IQ points [5].

Research on cohort differences in cognitive aging has mainly shown that later-born cohorts outperform earlier-born cohorts [3, 6–13]. The results depend on several factors, such as the cognitive domains assessed, participants’ age range, the number of years between birth cohorts and whether studies have examined cohort differences in cognitive performance trajectories [14]. In a more recent study, Brailean et al. [14] found that at the age 65 to 75 the later-born cohort (1931–1941) had better general cognitive performance, inductive reasoning and processing speed compared to the earlier-born cohort (1920–1930). The cohort differences in general cognitive performance and inductive reasoning were explained by better education in the later cohort, but processing speed was not. Furthermore, Christensen et al. [15] studied cohort differences in elderly Danish individuals and found that at age 95, a 1915 birth cohort scored significantly better on a battery of five cognitive tests and the Mini-Mental State Examination (MMSE) than a 1905 birth cohort assessed at age 93. The largest birth cohort differences were generally found on measures of fluid cognition that emphasize mental speed, abstraction and reasoning, whereas smaller birth cohort differences were found on measures of crystallized-like abilities that require general knowledge and vocabulary [13].

The results consistently favoring later-born cohorts, reported in several studies, may be explained by the Flynn effect or they may stem from differences in the age trajectories of cognitive abilities. Some studies have assessed whether the slopes of change differ between people aging at different times. The results have been mixed and thus the evidence on birth cohort differences in old age remains inconclusive [13]. Nevertheless, there is also some evidence that cohorts do not differ in age-related decline in verbal, spatial, memory or processing speed abilities [6] or in rates of change in psychomotor speed, category fluency and letter fluency [11].

Owing to the different historical time periods in which the present participants have lived their lives—Finland was a largely agrarian economy when the earlier cohorts were born in 1910–1915—the aim of this study was to evaluate possible birth cohort differences in the cognitive performance of older men and women born and assessed 28 years apart. The study design is unique in that it incorporates multiple highly relevant cognitive performance tests assessed with identical and highly comparable standardized measures. We compared cohort samples of participants who had been assessed either in 1989–1990 or in 2017–2018. A further aim was to study the factors underlying potential differences between the two cohorts.

## Methods

### Study design and participants

This cohort study implemented harmonized data from the two cross-sectional Evergreen projects conducted at the University of Jyväskylä, Finland. The first Evergreen [16] cohort data were collected in 1989–1990 and the Evergreen II data in 2017–2018 as part of the *Active Ageing – Resilience and external support as modifiers of disablement outcome (AGNES)* project [17, 18]. Samples for both projects were drawn from the Finnish Population Register based on birth year and place of residence. All community-living 75- and 80-year-old residents of the city of Jyväskylä formed the target group and were measured in the same laboratory by trained health care professionals using the same standardized cognitive and reaction time tests. Members of the Evergreen cohort were born in 1910 and 1914 and members of the Evergreen II cohort were born in 1938–1939 and 1942–1943.

### Recruitment

The Evergreen and Evergreen II recruitment procedures are comparable. Recruitment was as inclusive as possible. All persons in the targeted age groups who were living in the community in a non-institutional setting in the recruitment area and who consented to take part were included.

The City of Jyväskylä, i.e., the recruitment area, had expanded between the first and second Evergreen projects owing to mergers with neighboring municipalities. However, we targeted people whose addresses were within the previous city area or in similar adjacent areas, including urban areas and suburbs with apartment buildings and detached houses.

In the first Evergreen study, conducted in 1989–1990, potential participants were sent a letter informing them about the study and suggesting a time for a home interview. Those who declined were asked to report their reasons for non-participation and the reasons were documented. In Evergreen II, conducted in 2017–2018, potential participants were first sent a letter informing them about the study after which we enquired about their willingness to take part by telephone. For those willing to take part, a home interview was scheduled. During the phone call, those declining to take part were asked about their reasons for non-participation. In Evergreen, 500 (77%) and in Evergreen II, 726 (40%) of those eligible participated in the home interviews and research center assessments. Information on self-rated health was available for 47% of non-participants in the earlier cohort and for 73% of non-participants in the later cohort. The most common reason for non-participation at both times was lack of interest or not having time to take part. Poor health was a slightly more common reason in the earlier cohort, although self-rated health did not differ between the non-participants of the study cohorts. For an overview of the full design and further details on participation and non-participation, see Koivunen et al. [19].

### Functional assessments

In both projects, the implementation and assessment methods were identical. The interviews were conducted in the participants’ homes and the cognitive performance tests, as part of a more extensive study protocol, in the Health and Sport Laboratory of the Faculty of Sport and Health Sciences of the University of Jyväskylä. The laboratory environment and measurement equipment were similar for both birth cohorts.

### Outcome measures

#### Cognitive performance

The Digit Span test was used to measure auditory short-term memory. This test required verbal recall of forward and backward number series read to the participant by the examiner [20, 21]. The test started with a forward-series of four digits and progressed with additional digits up to a maximum of eight digits or until the participant failed twice. The same procedure was then repeated with backwards-series (from two to seven digits). The score was the number of correctly repeated digit spans in both the forward and backward tests (maximum 15). The forward digit span relies on the phonological loop of working memory, whereas the backward digit span also engages the central executive component [22].

The Digit Symbol coding task was used to measure processing speed and short-term visual memory (Wechsler Adult Intelligence Scale-Revised) [23]. The participant drew the correct symbols below their equivalent numbers by using a number-to-symbol coding key. The test time limit was 90 s. The score is the number of correct symbols in the correct order (maximum 65). Several abilities, such as processing speed, working memory, visuospatial processing and attention are needed to perform well in the task, and scores have been found to decline steeply with age [24, 25].

Phonemic verbal fluency, which is an indicator of semantic memory and verbal ability, was assessed with a modified version of the Word Fluency Test [21, 26]. Because the test task requires both clustering (certain phonemic categories) and the ability to switch efficiently to a new strategy, it is a sensitive indicator of brain dysfunction [27]. Participants were instructed to name as many Finnish words starting with the letter K as possible during 3 min (instead of the original 5 min). The total score is the number of acceptable words.

Reaction time, assessing motor and mental response speeds as well as response impulsivity and accuracy, was measured using a simple finger movement task, followed by a complex finger movement task using an apparatus constructed in-house at the University of Jyväskylä [28]. The participant was seated with the index finger of the dominant hand on the rest button in the middle of a row of buttons. The task was to move the finger as soon as possible onto the button closest to the light when it was switched on. First, the participant performed the simple reaction time test, in which the same light was switched on each time, and then a more complex test, where any one of seven lights was randomly switched on. Reaction time and movement time were measured in milliseconds. The simple and complex tasks were each repeated 12 times. The average times of the last five correctly performed tasks were recorded as the result.

### Covariates

Our analyses were not adjusted for any confounders. The age and sex groups of the different cohorts were similar, and we concluded that differences in the covariates between cohorts were more likely to be due to factors underlying cohort differences than to confounders. To study these factors, we chose correlates of cognitive performance that differed between the cohorts and that, in theory, can be part of the mechanism leading to secular change. Socio-economic position was indicated by years of full-time education. Physical activity was assessed with a single validated self-report question with six response options ranging from mostly sitting and resting to regular strenuous exercise [29]. For the statistical analysis, the responses were re-coded as low, moderate and high.

### Statistical methods

To compare the recent and earlier same-age cohorts, we used t-tests for continuous and chi-square test for categorical variables. Factors underlying potential cohort differences were studied in a set of linear regression models separately for sex and age. First, with birth cohort as an independent variable, the models were fitted with each cognitive test separately as a dependent variable. Subsequently, we ran several models, adding covariates one at a time to analyze which of them attenuated differences between the cohorts in cognitive performance.

## Results

All participant characteristics had statistically significant cohort differences in both sexes and age groups (Table 1). Years of education were twofold (*p* =  < 0.001) higher in the later-born than earlier-born cohort. Daily physical activity (*p* =  < 0.001) and self-rated health (p =  < 0.001) were also higher in the later-born cohort. The participation rate in the later study was lower. The reasons for non-participation have been reported in detail elsewhere [19]. In brief, we observed no explicit differences between the non-participants of the earlier and later cohorts, indicating the absence of systematic selection bias between the two Evergreen studies.

**Table 1:** Descriptive statistics and cohort differences between 75- and 80-year-old men and women in 1989–1990 (Evergreen cohort) and 2017–2018 (Evergreen II cohort)

	75 years		*p*-value	80 years		*p*-value
	1989–1990	2017–2018		1989–1990	2017–2018	
Men	*n* = 104	*n* = 183		*n* = 60	*n* = 132	
Years of education, mean (SD)	6.2 (3.5)	12.2 (4.4)	< .001	5.9 (4.1)	11.9 (4.4)	< .001
Height, cm, mean (SD)	169.5 (6.2)	172.7 (6.0)	< .001	169.1 (6.5)	172.3 (6.1)	.001
Weight, kg, mean (SD)	74.1 (10.7)	80.4 (13.0)	< .001	75.3 (12.8)	80.1 (12.6)	.017
Self-rated health, *n* (%)			< .001			< .001
Very good/good	13 (13)	106 (58)		10 (17)	60 (46)	
Average	79 (76)	72 (39)		35 (59)	69 (52)	
Poor/very poor	12 (12)	5 (3)		14 (24)	3 (2)	
Physical activity, *n* (%)			< .001			< .001
Low	30 (29)	10 (6)		24 (40)	15 (12)	
Moderate	62 (60)	127 (70)		33 (55)	94 (72)	
High	12 (12)	44 (24)		3 (5)	21 (16)	
Women	*n* = 191	*n* = 251		*n* = 145	*n* = 160	
Years of education, mean (SD)	6.1 (3.2)	12.0 (4.1)	< .001	5.7 (3.1)	11.8 (6.2)	< .001
Height, cm, mean (SD)	155.8 (5.6)	159.4 (5.1)	< .001	155.5 (5.4)	158.2 (5.5)	< .001
Weight, kg, mean (SD)	67.5 (11.6)	71.1 (12.4)	.002	64.5 (10.2)	69.7 (12.0)	< .001
Self-rated health, *n* (%)			< .001			< .001
Very good/good	27 (14)	137 (55)		18 (13)	67 (42)	
Average	139 (73)	108 (43)		93 (65)	85 (53)	
Poor/very poor	25 (13)	6 (2)		33 (23)	8 (5)	
Physical activity, *n* (%)			< .001			< .001
Low	42 (22)	29 (12)		48 (34)	21 (13)	
Moderate	139 (74)	190 (76)		92 (65)	120 (76)	
High	7 (4)	30 (12)		2 (1)	18 (11)	

Cohort differences in the cognitive performance measures by sex and age group are presented in Table 2. In men, statistically significant differences between the birth cohorts were found in the Verbal Fluency and WAIS Digit Symbol tests for both age groups (*p* =  < 0.015). A statistically significant difference was also found in the complex movement task in 75-year-old men (*p* = 0.049). In women, a statistically significant difference was found between the birth cohorts in each measured outcome (*p* =  < 0.004), except for the Digit Span total score in 80-year-olds. All the between-cohort differences in both men and women favoured the later-born cohort.

**Table 2:** Cohort differences in cognitive performance of 75- and 80-year-old men and women in 1989–1990 (Evergreen cohort) and 2017–2018 (Evergreen II cohort)

	75 years		Cohort difference*	*p*-value	80 years		Cohort difference*	*p*-value
	1989–1990	2017–2018			1989–1990	2017–2018		
	Mean (SD)	Mean (SD)			Mean (SD)	Mean (SD)		
Men	*n* = 104	*n* = 183			*n* = 60	*n* = 131		
Digit Span total score, number	9.3 (1.7)	9.6 (1.7)	0.3 (3%)	.135	9.2 (1.7)	9.4 (1.6)	0.2 (2%)	.346
Verbal fluency, number	29.9 (10.9)	33.6 (12.5)	3.7 (12%)	.015	22.6 (10.6)	33.3 (11.8)	10.7 (47%)	< .001
WAIS digit symbol test, number	21.9 (9.7)	32.5 (10.7)	10.6 (48%)	< .001	19.1 (9.3)	28.3 (8.5)	9.2 (48%)	< .001
Simple movement task, ms	654.9 (274.7)	617.9 (170.1)	37.0 (− 6%)	.240	693.1 (213.3)	648.3 (162.4)	44.8 (− 7%)	.116
Complex movement task, ms	1046.0 (710.6)	913.4 (294.2)	132.6 (− 13%)	.049	1091.3 (359.3)	1031.5 (391.7)	59.8 (− 6%)	.324
Women	*n* = 191	*n* = 251			*n* = 145	*n* = 160		
Digit Span total score, number	9.1 (1.7)	9.5 (1.6)	0.4 (4%)	.004	9.2 (1.4)	9.4 (1.7)	0.2 (2%)	.283
Verbal Fluency, number	29.3 (10.9)	37.5 (11.8)	8.2 (28%)	< .001	28.3 (13.7)	37.9 (12.2)	9.6 (34%)	< .001
WAIS Digit Symbol test, number	21.3 (9.5)	35.4 (9.8)	14.1 (66%)	< .001	18.8 (9.8)	31.9 (9.0)	13.1 (70%)	< .001
Simple movement task, ms	731.4 (293.7)	652.7 (135.7)	78.7 (− 11%)	.001	823.1 (416.1)	700.8 (226.5)	122.3 (− 15%)	.002
Complex movement task, ms	1131.3 (492.7)	1025.3 (313.6)	106.0 (− 9%)	.025	1260.4 (568.2)	1100.6 (313.5)	159.8 (− 13%)	.003

*Cohort difference = Absolute (relative) differences between the birth cohorts (%)

*p*-value = *t*-test

In men and 80-year-old women, the cohort differences were not statistically significant, and in 75-year-old women, the cohort differences were attenuated after adjustment for education (Tables 3, 4). We did not find clear cohort differences by sex or age in the Digit Span total score. Cohort differences in verbal fluency were generally attenuated after adjustment. Education, especially in 75-year-old men and women, and physical activity in 75-year-old men explained a substantial proportion of the cohort differences, rendering the associations statistically non-significant. No cohort differences by age were observed in the simple and only a small difference in the complex movement task in men. In women, however, education explained the cohort differences in both the simple and complex movement task in both age groups.

**Table 3:** Linear regression of the association between the birth cohort and cognitive performance measures in men

	Men 75 years			Men 80 years		
	Birth cohort	*p*-value	Model	Birth cohort	*p*-value	Model
	*β* (SE)		Adjusted *r*^2^	*β* (SE)		Adjusted *r*^2^
Digit Span total score						
Birth cohort	.31 (.21)	.135	.004	.24 (.25)	.346	.000
+ PA	.20 (.22)	.359	.005	.16 (.27)	.547	− .007
+ education	− .50 (.24)	.039	.089	− .42 (.29)	.153	.079
Verbal fluency						
Birth cohort	3.6 (1.5)	.015	.017	10.7 (1.8)	< .001	.154
+ PA	1.7 (1.5)	.259	.061	8.7 (1.9)	< .001	.196
+ education	− 3.2 (1.7)	.055	.152	6.5 (2.1)	.002	.212
WAIS digit symbol test						
Birth cohort	10.6 (1.3)	< .001	.195	9.2 (1.4)	< .001	.186
+ PA	8.6 (1.3)	< .001	.259	8.2 (1.5)	< .001	.206
+ education	3.0 (1.4)	.031	.359	4.9 (1.5)	.002	.291
Simple movement task						
Birth cohort	− 37.0 (31.5)	.240	.002	− 44.8 (28.4)	.116	.008
+ PA	12.2 (30.4)	.689	.143	− 6.4 (28.9)	.825	.083
+ education	− 7.0 (36.0)	.845	.013	− 8.2 (33.2)	.805	.028
Complex movement task						
Birth cohort	− 132.6 (67.0)	.049	.013	− 59.8 (60.5)	.324	.000
+ PA	− 25.4 (65.5)	.699	.142	− 2.3 (63.3)	.971	.030
+ education	− 134.7 (77.2)	.082	.009	− 17.7 (68.9)	.797	.009

*β* unstandardized beta, *SE* standard error, *PA* physical activity

**Table 4:** Linear regression of the association between the birth cohort and cognitive performance measures in women

	Women 75 years			Women 80 years		
	Birth cohort	*p*-value	Model	Birth cohort	*p*-value	Model
	*β* (SE), *p*		Adjusted *r*^2^	*β* (SE), *p*		Adjusted *r*^2^
Digit Span total score						
Birth cohort	.46 (.16)	.004	.017	.20 (.18)	.284	.001
+ PA	.44 (.16)	.006	.011	.13 (.19)	.490	− .003
+ education	− .28 (.19)	.144	.097	− .32 (.23)	.152	.035
Verbal Fluency						
Birth cohort	8.2 (1.1)	< .001	.111	9.6 (1.5)	< .001	.119
+ PA	7.8 (1.1)	< .001	.122	8.7 (1.6)	< .001	.114
+ education	1.3 (1.3)	.320	.235	3.6 (1.8)	.044	.192
WAIS Digit Symbol test						
Birth cohort	14.1 (0.9)	< .001	.341	13.1 (1.1)	< .001	.326
+ PA	13.7 (1.0)	< .001	.342	12.5 (1.2)	< .001	.320
+ education	6.8 (1.0)	< .001	.492	7.2 (1.3)	< .001	.426
Simple movement task						
Birth cohort	− 78.7 (24.0)	.001	.030	− 122.3 (38.1)	.001	.030
+ PA	− 68.9 (23.8)	.004	.086	− 82.4 (39.7)	.039	.057
+ education	− 50.8 (29.5)	.086	.034	− 48.3 (43.3)	.266	.036
Complex movement task						
Birth cohort	− 106.0 (47.1)	.025	.012	− 159.8 (52.4)	.002	.027
+ PA	− 92.1 (47.5)	.053	.043	− 117.1 (54.7)	.033	.038
+ education	− 42.6 (57.7)	.461	.020	− 64.6 (64.7)	.319	.036

*β* unstandardized beta, *SE* standard error, *PA* physical activity

## Discussion

Our objective in this study was to assess birth cohort differences in cognitive performance in older men and women who have lived through different historical time periods. To do so, we compared population-based samples of 75- and 80-year-old people assessed either in 1989–1990 (Evergreen project) or 2017–2018 (Evergreen II project). The results provide strong evidence that older people today have better cognitive abilities compared to counterparts measured 28 years earlier. The results are also in line with earlier findings that later-born cohorts outperform earlier-born cohorts in cognitive abilities, e.g. [3, 6, 9, 13]. The present results are unique as they derive from multiple highly relevant cognitive performance tests assessed with identical and highly comparable standardized measures in two comparable cohorts examined almost 3 decades apart. These results provide us with novel information about differences in cognitive performance in people growing old during different historical periods.

Finland was largely agrarian economy when the earlier cohorts were born in 1910–1915. Children at that time worked from an early age, lived through the Civil War in 1918 and served as young adults in the Second World War. The later cohort was born in 1938–1942. Although they lived their early childhood during war-time (i.e., 1939–1944), they had more propitious life course exposures that positively affected their health and functioning, as many reforms were introduced in the immediate post-war years. The foundations of the Nordic welfare system were laid in the 1930s, including the provision of free school meals for all children and longer obligatory education. Finland developed rapidly in the 1950s, access to secondary and tertiary education improved and the female disadvantage in education narrowed [30]. This partially explains our findings, which showed a doubling in the length of education between the earlier and later cohorts. Increased educational attainment during the twentieth century has been associated with better cognitive performance in midlife and old age [3], supporting the findings of our study. Thus, education can be expected to be a strong determinant of the higher levels of work complexity in later-born cohorts and, therefore, a strong predictor of higher later life cognition in these cohorts [3]. Many of the birth cohort effects remained unexplained by the variables available in our data. Some could, at least partly, be explained by improved medical care and better access to health care. However, we can probably rule out genetic differences between the cohorts as an explanation, as there has been little in-migration in Finland since the resettlement of the Karelian population during the Second World War. Overall, in both sexes, better cognitive performance was partially explained by longer education and a higher level of physical activity. The factors that shape and maintain cognitive performance are known to differ across cohorts and may stem from several societal and environmental changes [12, 31]. These include higher educational attainment and improved health care [3], population movement from rural to urban areas and smaller families [7], improved nutrition [32] and hygiene [7], more complex and stimulating work [33], as physical and mixed types of work have been shown to be detrimental for cognitive functioning and scores on the Work Ability Index [34], cognitively stimulating leisure time activities [35], social engagement [36] and changes in processing from more characteristically verbal to more iconic representations due to the rise of visually oriented modalities in film, television, computer games, and other media [7] and, more recently, social media and mobile devices. In addition, the benefits of physical activity on cognitive performance have been shown in many studies [37, 38]. In this study, physical activity explained a substantial portion of the cohort differences in the simple and complex movement tasks, especially in 75-year-old men. Many of these factors favor later-born cohorts and likely drive the Flynn effect [3], thereby offering several explanations for the current results. However, it is worth noting, that our participants were born around the years when the initial studies on children’s intelligence were initiated, and later led to launching of the concept “Flynn effect”. Among these early cohorts, the “Flynn effect” appears to hold even though studies in more recent cohorts suggest stagnation or even decline [39, 40]. Furthermore, the selection of the study participants differs between the earlier- and later-born cohorts due to mortality. Because the war-time mortality rate was higher and owing to selective mortality, particularly with respect to cognitive functions, the Evergreen I sample represents a much more selected population than the Evergreen II sample. This, however, only increases the significance of the improved level of cognitive function observed in the later-born cohort.

Karlsson et al. [3] investigated three population-based samples born in 1901, 1906, and 1930 and found substantial differences in level of cognitive performance where later-born cohorts outperformed earlier born cohorts. They expected gender to be less important as a moderator of the level and change in cognitive performance in the later-born cohorts given that gender had more influence on educational and social opportunities in the earlier-born cohorts. In our study, the 75-year-old women and men in the earlier-born cohort performed similarly in the Digit Span, Verbal Fluency and WAIS Digit Symbol tests, whereas in the later-born cohort women out-performed men in the Verbal Fluency and WAIS digit symbol test. This was also true for the 80-year-olds, except for the Verbal Fluency test where, in the earlier-born cohort, women outperformed men. This testifies to the rise in the educational and social level of women in particular, and can be at least partly explained by the fact that in the earlier-born cohorts most women were stay-at-home housewives whereas in the later-born cohort gender roles had changed and women’s participation in the labor force had increased [10]. Interestingly, Rönnlund and Nilsson [41] pointed out that Emanuelsson and Svensson [42] and Emanuelsson et al. [43] observed a widening gender gap (i.e., an increasing female advantage in the semantic factor over time) among 13-year-old children, showing that girls were even more ahead of boys in 1990 than in 1960. Rönnlund et al. 2008 [41] speculated, that based on the trend for Swedish women to show higher levels of educational attainment than men, a significant gender-by-time interaction will be found in future studies. In this study, men, in turn, out-performed women in simple reaction and movement time tests irrespective of time or age.

The societal implications for cognitive aging of systematic and substantial positive trends in cohort levels and in cohort changes are considerable [10]. Research has shown that we are living not only longer, but also healthier, lives. It has been reported that today’s 70-year-olds are as healthy as 60-year-olds [44] or 65-year-olds [7] were 30 years ago. Positive cohort differences in abilities such as reasoning suggest that people can be productively employed much longer in professions that require strong reasoning skills [45]. We have shown this same phenomenon for functional capacity (i.e. walking speed, grip strength and knee extension strength) in a recent study with 75 and 80-year-olds [19].

One of the main strengths of the study is that our data are based on representative population-based samples that allowed us, using the same standardized cognitive performance tests, to compare, at the same chronological ages (i.e., 75 and 80), two birth cohorts of older men and women born up to 28 years apart. Furthermore, participant recruitment was comparable in both cohort studies and non-participant characteristics did not differ between the studies, further supporting the comparability of the cohorts. Our study is also unique in the length of the interval, almost three decades, between the two studies.

This study has its limitations. The participation rate was lower in the more recent than earlier study, which could mean that the participants in the more recent study represent a healthier section of the target population than counterparts in the earlier study. However, since the non-participants did not differ between the studies, we may assume that the cohorts were comparable and that the lower participation rate in the later study did not lead to biased results. Another possible limitation is that cohort differences in comorbidity could not be included in the analyses due to changes in the diagnostics, treatment and recording of chronic conditions over the past three decades. Moreover, the results may be unique to Finland; however, it is likely that they can be generalized to other countries that have undergone similar societal changes during the last 100 years. We do not have data on life-course exposures earlier in the participants’ lives; this information would have strengthened our conclusions on the possible reasons for the differences between the cohorts. Finally, these finding are based on assessments made at two time points, and therefore it is not possible to test the linearity of change over time.

In conclusion, this study provides strong evidence of better overall cognitive performance in the more recent birth cohorts of 75- and 80-year-old Finnish older people. The cognitive performance measures used underlie traits such as memory, attention, processing speed and problem solving.
